# Mecânica Atrial na Cardiomiopatia Hipertrófica: Discriminando Hipertrofia de Fibrose Ventricular

**DOI:** 10.36660/abc.20200890

**Published:** 2021-11-17

**Authors:** Patrícia Marques-Alves, João André Ferreira, André Azul Freitas, José Paulo Almeida, Rui Baptista, Graça Castro, Rui Martins, Paulo Donato, Maria João Ferreira, Lino Gonçalves

**Affiliations:** 1 Departamento de Cardiologia Centro Hospitalar e Universitário de Coimbra Coimbra Portugal Departamento de Cardiologia , Centro Hospitalar e Universitário de Coimbra , Coimbra – Portugal; 2 Instituto de Investigação Clínica e Biomédica de Coimbra Universidade de Coimbra Coimbra Portugal ICBR Instituto de Investigação Clínica e Biomédica de Coimbra , Universidade de Coimbra , Coimbra – Portugal; 3 Faculdade de Medicina Universidade de Coimbra Coimbra Portugal Faculdade de Medicina , Universidade de Coimbra , Coimbra – Portugal; 4 Departamento de Radiologia Centro Hospitalar e Universitário de Coimbra Coimbra Portugal Departamento de Radiologia , Centro Hospitalar e Universitário de Coimbra , Coimbra – Portugal

**Keywords:** Cardiomiopatia Hipertrófica, Hipertensão, Ecocardiografia/métodos, Espectroscopia de Ressonância Magnética/métodos, Hipertrofia Ventricular Esquerda

## Abstract

**Fundamento:**

A cardiomiopatia hipertrófica (CMH) e a hipertrofia ventricular esquerda (HVE) secundária à hipertensão arterial sistêmica (HAS) podem estar associadas a anormalidades funcionais do átrio esquerdo (AE).

**Objetivos:**

Caracterizar a mecânica do AE na CMH e na HAS e avaliar qualquer correlação com a extensão da fibrose ventricular esquerda medida por ressonância magnética cardíaca (RMC) em pacientes com CMH.

**Métodos:**

A função longitudinal do AE derivada do ecocardiograma bidimensional com *speckle tracking* foi adquirida a partir de cortes apicais de 60 pacientes com CMH e 34 indivíduos controles, pareados por idade. Pacientes com CMH também foram submetidos à RMC, com medida da extensão do realce tardio por gadolínio. A associação com parâmetros de *strain* do AE foi analisada. Valores p < 0,05 foram definidos como estatisticamente significativos.

**Resultados:**

A média da fração de ejeção do ventrículo esquerdo não foi diferente entre os grupos. A razão E/e’ estava comprometida no grupo CMH e preservada no grupo controle. A mecânica do AE estava significativamente reduzida na CMH em comparação aos pacientes com HAS. O *strain rate* do AE nas fases de reservatório (SRrAE) e na fase contrátil (SRctAE) foram os melhores parâmetros de discriminação de CMH com uma área sob a curva (AUC) de 0,8, seguido do *strain* do AE na fase de reservatório (SrAE) (AUC 0,76). O SRrAE e o SRctAE apresentaram elevada especificidade (89% e 91%, respectivamente), e o SrAE apresentou sensibilidade de 80%. Um decréscimo de 2,79% no strain rate do AE na fase de condução (SRcdAE) foi preditor de um aumento de 1 cm na extensão do RT pelo gadolínio (r^2^=0,42, β 2,79, p=0,027).

**Conclusões:**

O SRrAE e o SRctAE foram os melhores fatores de discriminação de HVE secundária à CMH. O SRcdAE foi preditor do grau de fibrose ventricular esquerda avaliada por RMC. Esses achados sugerem que a mecânica do AE pode ser um potencial preditor de gravidade de doença na CMH.

## Introdução

A hipertrofia ventricular esquerda (HVE), presente na cardiomiopatia hipertrófica (CMH) e na hipertensão arterial sistêmica (HAS), está geralmente relacionada à disfunção do miocárdio e risco aumentado de morte súbita.^1 , 2^ Na HAS, a HVE ocorre como uma resposta à sobrecarga de pressão e, na CMH, um complexo processo de remodelamento é iniciado como uma resposta de componentes de cardiomiócitos e não-cardiomiócitos a estímulos mecânicos dinâmicos e neuro-humorais.^1 - 3^ A CMH é um distúrbio autossômico dominante, associado a mutações em genes sarcoméricos, que afeta o miocárdio atrial e ventricular.^1 , 2^

O exame de ressonância magnética cardíaca (RMC) permite uma descrição abrangente de HVE e fibrose relacionadas à CMH, por meio da técnica de realce tardio (RT) pelo gadolínio.^4^ Esse método, usado na avaliação quantitativa do ventrículo esquerdo (VE), permite a caracterização dos estágios da CMH, do remodelamento e da disfunção sistólica do VE, e é um importante preditor de morte súbita.^4 , 5^ A disfunção sistólica ocorre comumente em estágios terminais da CMH, e uma parcela significativa dos pacientes apresentam algum grau de disfunção diastólica.^2 , 6^

Aumento da massa ventricular esquerda e disfunção diastólica estão associados a dilatação e disfunção progressivas do átrio esquerdo (AE). Além disso, o remodelamento do AE é uma característica comum tanto da CMH como da HAS.^2 , 7^ Ainda, o tamanho e o volume do AE mostraram-se fatores determinantes da capacidade no exercício^8^ e de eventos adversos maiores cardíacos e cerebrovasculares em pacientes com CMH.^9^

Uma vez que o AE está relacionado com o desempenho do VE dadas as suas funções de reservatório durante a sístole ventricular, condução durante a diástole ventricular precoce, e bombeamento durante a diástole ventricular tardia, a miopatia do AE poderia estar associada a desfechos independentemente da função do VE.^10^ A função do VE correlaciona-se com sintomas da insuficiência cardíaca na CMH e é forte preditora do desenvolvimento de fibrilação atrial (FA).^11 , 12^

A avaliação da mecânica do AE por meio do ecocardiograma bidimensional com *speckle tracking* (2D-STE) tem se mostrado um marcador viável e reprodutível para a função do AE.^13 , 14^

Apesar de a HVE parecer ser o principal fator de disfunção na mecânica do VE,^2^ o grau de disfunção do AE em diferentes estados de HVE (em particular, HVE secundária à HAS e à CMH) não é totalmente entendido. Tanto a hipertrofia como a fibrose ventricular esquerda representam substratos da disfunção diastólica, e podem estar associadas à disfunção do VE na CMH. Assim, o presente estudo teve como objetivo (1) caracterizar os mecanismos do AE em pacientes com CMH e HAS com HVE significativa e (2) correlacionar a função do AE com fibrose do VE avaliada por RMC em pacientes com CMH.

## Métodos

### População do estudo

Este estudo observacional retrospectivo incluiu 60 pacientes diagnosticados com CMH (diagnóstico confirmado por RMC) e 60 pacientes hipertensos recrutados no ambulatório de nosso departamento. Excluímos pacientes com CMH obstrutiva, com janela acústica inadequada, FA identificada no eletrocardiograma basal, doença valvular grave ou moderada, doença cardíaca isquêmica, ou hipertensão pulmonar definida como pressão sistólica da artéria pulmonar (PSAP) > 45 mmHg. Os pacientes foram diagnosticados com HAS cerca de 4,2 ± 2,3 anos antes. Como controle, incluímos 34 pacientes sadios sem HAS, FA ou doença valvular, pareados por idade com pacientes com CMH e HAS.

### Procedimentos do estudo

Nós analisamos dados clínicos e ecocardiográficos de participantes divididos nos grupos CMH, HAS e controle. Dados de RMC dos pacientes com CMH no momento do diagnóstico também foram avaliados. Imagens ecocardiográficas foram coletadas 43±18 dias após o diagnóstico de CHM por RMC. O estudo foi aprovado pelo comitê científico e pelo comitê de bioética do Centro Hospitalar e Universitário de Coimbra (Coimbra, Portugal), e foi conduzido de acordo com a Declaração de Helsinki.

### Dados ecocardiográficos

Foi realizado um estudo bidimensional completo em todos os participantes, incluindo 2D-STE do VE e do AE com análise do *strain* longitudinal global (SLG). Utilizamos um aparelho de ultrassom cardiovascular Vivid 7 (GE Healthcare, Horten, Noruega) com um transdutor de imagem harmônica tecidual. Cortes ecocardiográficos padrões foram obtidos com otimização da taxa de quadros (60-80 fps em imagens 2D). Realizamos uma análise *offline* dos dados ecocardiográficos utilizando um programa específico (EchoPAC 16.0; GE Healthcare).

### Dimensões e função do VE

A avaliação do tamanho e da função sistólica do VE, incluindo a medida da fração de ejeção do VE (FEVE), diâmetro diastólico final do VE (DDFVE), e diâmetro sistólico final do VE (DSFVE), seguiu as recomendações atuais.^15^ O SLG do VE, derivado do EST, foi obtido usando um modelo de segmentação (em 16 segmentos) do VE.^16^ Foi também avaliada a função diastólica, incluindo velocidade E mitral, velocidade A mitral, e razão E/’ média.

### Imagens de deformação do AE

**Figura 1:**
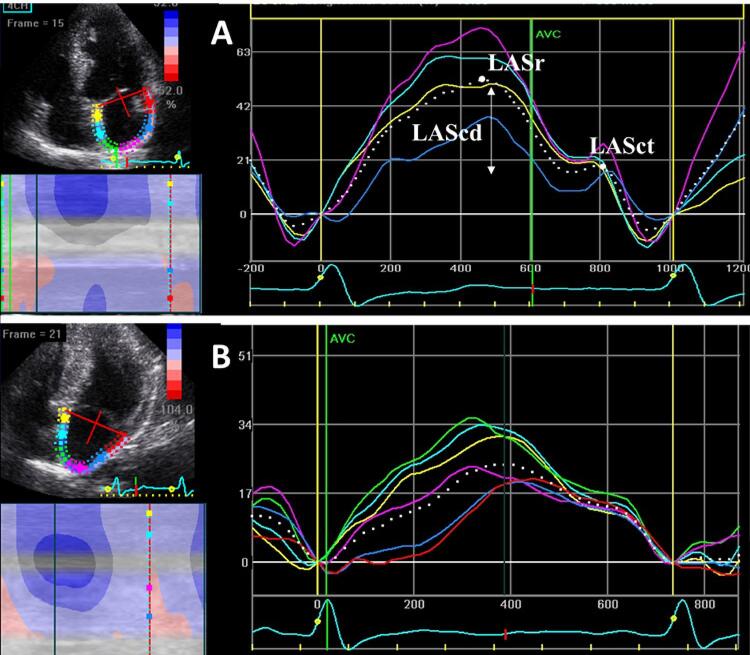
– Análise do strain do átrio esquerdo nos indivíduos controle (A) e pacientes com miocardiopatia hipertrófica (MCH) (B). Nos controles: strain do átrio esquerdo na fase de reservatório (SrAE) = 53%; strain do átrio esquerdo na fase de contração (SctAE); ScdAE: strain do átrio esquerdo na fase de condução (diferença entre SrAE e SctAE) = 21% (A). Nos pacientes: SrAE = 24%; SctAE = 14% e ScdAE = 10% (B)

A análise da mecânica do AE por 2D-STE foi realizada conforme recomendado previamente,^17 , 18^ com análise *offline* das curvas do *strain* longitudinal de cada segmento atrial usando um programa específico^17 , 18^ ( Figura 1 ). Para o processamento, o quadro (frame) inicial foi escolhido como aquele que refletisse o início da onda P. O *strain* e o *strain rate* do AE na sístole (SrAE e SRrAE, respectivamente), no início da diástole (ScdAE e SRcdAE), e no final da diástole (SctAE e SRctAE) foram medidos como indicadores das funções de reservatório, condução, e contração do AE, respectivamente.^14 , 18^

### Variabilidade entre observadores e intraobservador

Para análise da reprodutibilidade entre observadores, as medidas de *strain* e *strain rate* por 2D-STE, obtidas de 37 pacientes com CMH selecionados aleatoriamente, foram realizadas por um segundo investigador (JAF) e comparadas com aquelas do primeiro observador (PMA).

O primeiro observador repetiu as medidas nos mesmos 37 participantes, e a reprodutibilidade intraobservador foi então avaliada. Os observadores avaliaram diferentes regiões de interesse do AE e desconheciam (eram cegos a) as medidas anteriores.

### Ressonância magnética cardíaca

Todos os 60 pacientes com CMH foram submetidos à RMC, realizada com aparelhos de 1.5 T (Philips, Best, Holanda), utilizando-se protocolos padrões, como sugerido previamente.^4^ As imagens de RT com gadolínio foram adquiridas 10-20 minutos após administração endovenosa do contraste, como recomendado.^2^ A quantificação do RT foi realizada por ajuste manual do limiar de escala de cinza, para definir áreas de RT identificadas visualmente nos planos do eixo-curto e medidas em centímetros de extensão.

### Análise estatística

A normalidade da distribuição das variáveis contínuas foi avaliada por análise do histograma e o teste de Kolmogorov-Smirnov. As variáveis contínuas foram expressas em média ± desvio padrão, e as variáveis categóricas expressas em frequência (porcentagem). Diferenças entre grupos foram avaliadas pela análise de variância (ANOVA) *one-way* . Após rejeitar-se a hipótese nula, o teste de comparação múltipla de Bonferroni foi realizado. Para cada variável com distribuição não normal, a homogeneidade da variância foi avaliada utilizando o teste de Levene. Para as variáveis categóricas, utilizou-se o teste do qui-quadrado ou o teste exato de Fisher, conforme apropriado. Regressão linear foi usada para correlacionar vários parâmetros contínuos. Pressupostos para a regressão linear foram verificadas antecipadamente – relação linear entre os dados foi visualmente avaliada por gráficos de dispersão; não foram detectados *outliers* ; a autocorrelação foi excluída pelo teste de Durbin-Watson; a homogeneidade da variância dos resíduos (homoscedasticidade) também foi verificada visualmente em gráficos de resíduos versus valores ajustados. Análise de curvas da Característica de Operação do Receptor (curvas ROC) foi realizada para se estimar o poder discriminatório dos parâmetros da mecânica do AE na CMH versus HA. As curvas foram comparadas pelo método Delong. Para a avaliação da variabilidade entre observadores e intraobservador das medidas de *strain* e *strain rate* foram utilizados método Bland-Altman, coeficiente de correlação intraclasse, e coeficiente de variação. Valores p < 0,05 (teste bicaudal) foram considerados como estatisticamente significativos. As análises estatísticas foram realizadas utilizando o programa Stata IC para Windows (versão 13; StataCorp, Lakeway Drive, TX, EUA). Em relação ao tamanho amostral, planejamos um estudo de uma variável de resposta contínua em pacientes e controles independentes, na proporção de 0,5 indivíduos controle por paciente. Na análise da mecânica do AE ( *strain* do na fase de reservatório e na fase de contração), a resposta desejável em cada grupo é normalmente distribuída com um desvio padrão de cinco.^7 , 10^ Se a diferença entre pacientes e controle fosse quatro, necessitaríamos de 51 pacientes e 26 controles para conseguirmos rejeitar a hipótese nula de que as médias populacionais dos grupos experimental e controle fossem iguais com uma probabilidade (poder) de 0,9. A probabilidade de se cometer um erro do tipo I associado com esse teste com essa hipótese nula é de 0,05. Poderíamos ainda incluir 60 pacientes com CMH e 60 pacientes com HAS para melhorar o poder estatístico do estudo.

## Resultados

### População do estudo

As características clínicas da população do estudo estão resumidas na Tabela 1 . A idade média dos pacientes com CMH foi de 55 ± 18 anos, e 57% dos pacientes eram do sexo masculino. Esse dado não foi significativamente diferente do grupo de pacientes com HAS e controles. Os pacientes com HAS apresentaram mais diabetes mellitus, dislipidemia e obesidade.

**Tabela 1:** – Características clínicas e demográficas da população estudada

Característica	Controles (n=34)	Grupo CMH (n=60)	Grupo HAS (n=60)	Valor-p
Idade, anos (±SD)	56±10	55±18	61±12	0,081*
Sexo masculino (%)	20 (55)	34 (57)	34 (57)	0,124^#^
Diabetes *mellitus* (%)	0	15 (25)	35 (58)	0,022^#^
Dislipidemia (%)	0	24 (40)	43 (72)	0,014^#^
Obesidade (%)	0	12 (20)	28 (47)	0,031^#^
**Medicamentos anti-hipertensivos**				
Diuréticos (%)	0	15 (25)	32 (53)	0,018^#^
Betabloqueadores	0	45 (75)	37 (62)	0,068^#^
IECA/BRA	0	41 (68)	45 (75)	0,072^#^
BCC	0	27 (45)	42 (70)	0,032^#^
Outros	0	12 (20)	20 (34)	0,056^#^

Dados apresentados como média ± desvio padrão ou frequência (porcentagem). IECA: inibidores da enzima conversora de angiotensina; BRA: bloqueadores de receptores da angiotensina; BCC: bloqueadores de canais de cálcio; CMH: cardiomiopatia hipertrófica; HAS: hipertensão arterial sistêmica. * ANOVA one-way. # Teste do qui-quadrado.

No grupo com CHM, observou-se um uso maior de betabloqueadores, enquanto no grupo com HAS, observou-se uma maior prescrição de inibidores da enzima conversora de angiotensina (IECA) e bloqueadores de receptores da angiotensina (BRA).

### Parâmetros ecocardiográficos convencionais

As características ecocardiográficas dos participantes estão resumidas na Tabela 2 . A média da FEVE não foi diferente entre os grupos. O DFVE foi maior no grupo com HAS que no grupo com CMH. A PSAP não foi diferente entre os grupos CHM e HAS, e estava significativamente reduzida no grupo controle.

**Tabela 2:** – Parâmetros ecocardiográficos da população estudada

Parâmetros	Controles	Grupo HAS	Grupo CMH	p-valor global	p-valor: controles vs HAS	p-valor: CMH vs HAS
FEVE, %	62,9±4,3	62,9±4,9	66,5±10,1	0,083	0,969	0,055
DDFVE, mm	48,3±5,2	51,9±0,8	49,4±1,0	0,108	0,019	0,083
DSFVE, mm	30,3±3,2	32,3±0,7	30,7±0,9	0,369	0,119	0,225
SIV, mm	10,2±2,8	14,3±3,6	16,5±5,4	0,028	<0,001	0,032
SLG-VE, %	-20,6±1,1	-17,5±0,7	-12,7±0,5	<0,001	0,192	0,008
PSAP, mmHg	22,1±4,7	26,3±0,2	28,6±1,3	0,021	0,009	0,245
VAEi, mL/m^2^	23,5±4,2	31,1±1,3	33,5±2,5	<0,001	0,001	0,067
Velocidade E mitral, m/s	0,8±0,1	0,7±0,2	0,8±0,2	0,156	0,068	0,182
Velocidade A mitral, m/s	0,5±0,1	0,8±0,2	0,7±0,3	0,005	<0,001	0,151
Razão E/e’	7,0±1,65	13,2±1,2	16±1,0	<0,001	<0,001	0,035
SrAE, %	36,9±10,8	24,4±8,2	17,2±9,0	<0,001	<0,001	<0,001
ScdAE, %	25,9±13,3	19,9±8,7	15,4±9,1	<0,001	0,067	0,022
SctAE, %	10,9±6,2	5,1±0,9	1,9±0,3	<0,001	0,003	<0,001
SRrAE, %	1,9±0,5	1,2±0,1	0,8±0,1	<0,001	<0,001	<0,001
SRcdAE, %	-2,1±0,6	-1,8±0,1	-0,6±0,1	<0,001	0,082	<0,001
SRctAE, %	-1,9±0,7	-1,7±0,1	-0,9±0,1	<0,001	0,344	<0,001

*Análise estatística: one-way ANOVA com teste de Bonferroni para comparações múltiplas; CMH: cardiomiopatia hipertrófica; HAS: hipertensão arterial sistêmica; SIV: septo interventricular; SrAE: strain do átrio esquerdo na sístole (função de reservatório); ScdAE: strain do átrio esquerdo na diástole precoce (função de condução); SctAE: strain do átrio esquerdo na diástole tardia (função de contração); VAEi: volume do átrio esquerdo indexado; DDFVE: diâmetro diastólico final do ventrículo esquerdo; DSFVE: diâmetro sistólico final do ventrículo esquerdo; FEVE: fração de ejeção do ventrículo esquerdo; SLG-VE: strain longitudinal global; PSAP: pressão sistólica da artéria pulmonar.*

Em relação à função diastólica do VE, a velocidade mitral E não variou entre os grupos, e a velocidade A foi maior no grupo HAS. Valores anormais da razão E/e’ foram encontrados no grupo com CMH e, no grupo controle, essa razão estava preservada.

### Função do AE

**Figura 2:**
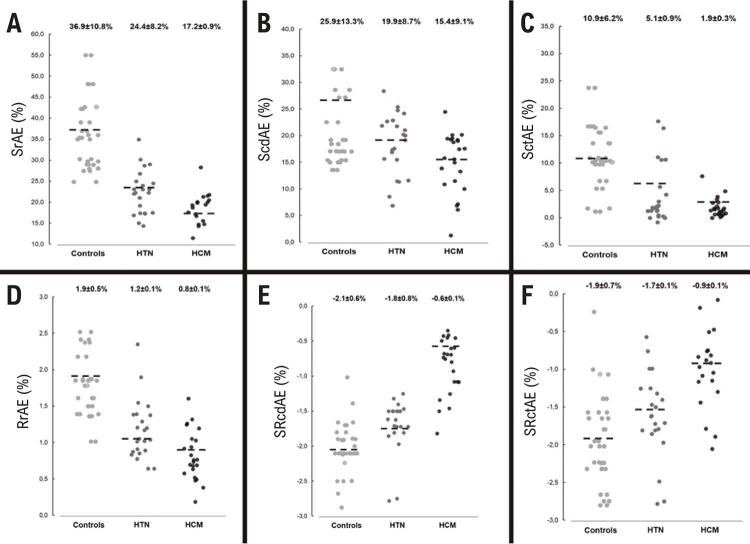
– Parâmetros de deformação do átrio esquerdo nos pacientes com cardiomiopatia hipertrófica (CMH), pacientes com hipertensão arterial sistêmica (HAS) e controles. Strain (A) e strain rate (D) na fase de reservatório(A); strain (B) e strain rate (E) na fase de condução; e strain (C) e strain rate (E) na fase de contração; SrAE: strain do átrio esquerdo na sístole (função de reservatório); ScdAE: strain do átrio esquerdo na diástole precoce (função de condução); SctAE: strain do átrio esquerdo na diástole tardia (função de contração); SRrAE: strain rate do átrio esquerdo na sístole (função de reservatório); SRcdAE: strain rate do átrio esquerdo na diástole precoce (função de condução); SRctAE: strain rate do átrio esquerdo na diástole tardia (função de contração).

Em comparação aos controles, os pacientes com CHM e HAS apresentaram valores de volume do AE indexado à área da superfície corporal (VAEi) significativamente maiores ( Tabela 2 ). Os parâmetros de deformação do AE estavam reduzidos nos grupos HAS e CMH em relação ao grupo controle. No grupo de pacientes com HAS, a função de reservatório estava preservada, apesar de significativamente reduzida em comparação aos controles; a fase de condução não foi diferente do grupo controle, e o *strain* (mas não o *strain rate* ) na fase de contração estava alterado nos pacientes com HAS ( Tabela 2 , Figura 2 ).

Todas as fases de deformação do AE estavam significativamente reduzidas no grupo CMH que no grupo HAS ( Tabela 2 , Figura 2 ). De todos os parâmetros da mecânica do AE, o SRrAE e o SRctAE foram os melhores discriminantes de CMH (versus HAS), seguido de SrAE. O SRrAR, e o SRctAE apresentaram elevada especificidade e alto valor preditivo positivo (VPP) ( Tabela 3 ). Apesar do baixo poder discriminatório, o ScdAE apresentou a maior especificidade (94%) e o SctAE a maior sensibilidade (95%) ( Tabela 3 ).

**Tabela 3:** – Poder discriminatório dos parâmetros ecocardiográficos obtidos de pacientes com cardiomiopatia hipertrófica e pacientes com hipertensão arterial sistêmica

	AUC	IC95%	Valor p	Sensibilidade (%)	Especificidade (%)	Critérios	VPP (%)	VPN (%)
SrAE, %	0.76	0.66-0.84	<0.001	80	71	21.8	73	78
ScdAE, %	0.65	0.54-0.74	0.012	32	94	9.9	84	58
SctAE, %	0.65	0.54-0.75	0.016	95	34	5.1	59	87
SRrAE, %	0.80	0.71-0.88	<0.001	65	89	0.8	86	72
SRcdAE, %	0.69	0.59-0.79	<0.001	54	87	-0.8	81	65
SRctAE, %	0.80	0.71-0.88	<0.001	64	91	-0.9	88	72
SIV (mm)	0.62	0.51-0.70	0.012	55	74	15.2	68	63
SLG-VE (%)	0.74	0.64-0.83	<0.001	57	84	-13.5	78	66
Razão E/e’	0.67	0.55-0.78	0.009	67	71	13	70	68

*AUC, área sob a curva; HAS: hipertensão arterial sistêmica; CMH: cardiomiopatia hipertrófica; SIV, septo interventricular; SrAE: strain do átrio esquerdo na sístole (função de reservatório); ScdAE: strain do átrio esquerdo na diástole precoce (função de condução); SctAE: strain do átrio esquerdo na diástole tardia (função de contração); SLG-VE: strain longitudinal global; VPN: valor preditivo negativo; VPP: valor preditivo positivo.*

### Parâmetros da RMC

Todos os pacientes com CMH foram submetidos à RMC (método padrão-ouro para o diagnóstico). O volume diastólico final indexado (VDFi) médio foi 96±32 mL/m^2^, a espessura média do septo interventricular (SIV) foi 18,7±3,5 mm. Aproximadamente 34% dos pacientes apresentaram movimento sistólico anterior da valva mitral, e 12% dos pacientes apresentaram CMH apical. RT com gadolínio foi observado em 52 (87%) dos pacientes com CHM, e a média da área de extensão foi de 2,8 cm.

### Parâmetros de RMC e ecocardiograma

**Figura 3:**
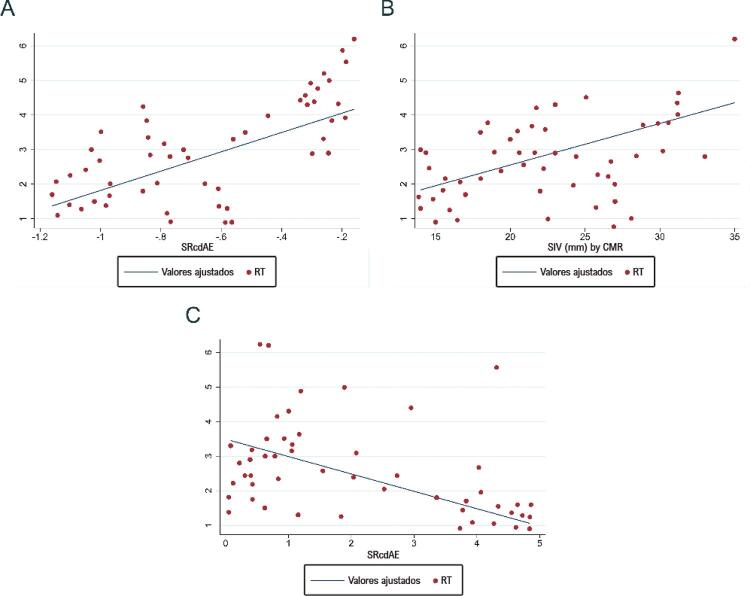
– Correlação linear entre extensão do realce tardio (RT) por gadolínio e espessura do septo interventricular (SIV) medida por ressonância magnética cardíaca (RMC) (A), strain do átrio esquerdo na diástole tardia (função de contração) (SctAE) (B) strain rate do átrio esquerdo na diástole precoce (função de condução) (SRcdAE) (C).

A Tabela 4 resume os resultados da análise de regressão linear entre a extensão do RT com gadolínio (cm) e vários parâmetros da RMC e do ecocardiograma em pacientes com CMH. As medidas do VE, VDFi (por RMC), DDFVE e DSFVE por ecocardiograma, o SLG do VE ou a razão E/E’ não foram preditores da extensão de RT. A espessura do SIV, medida por RMC, mas não por ecocardiografia, foi preditora do RT pelo gadolínio. Tanto o SrAE como o SRcdAE foram preditores da extensão de RT pelo gadolínio. Uma redução de 0,5% no SrAE e de 2,79% no SRcdAE foram preditores de um aumento de 1 cm no RT pelo gadolínio ( Figura 3 ).

**Tabela 4:** – Análise de regressão linear entre a extensão do realce tardio por gadolínio (cm) e parâmetros da ressonância magnética cardíaca (RMC) e de ecocardiografia

Realce tardio por gadolíno	Adj R^2^	β	Valor p
SIV por RMC	0,32	0,12	0,051
SIV por ecocardiografia	0,24	0,08	0,088
VDFi por RMC	0,01	0,01	0,843
DDFVE	0,01	-0,02	0,795
Razão E/E’	0,01	-0,04	0,802
SLG-VE	0,04	0,08	0,467
SrAE	0,12	-0,02	0,085
ScdAE	0,15	-0,01	0,092
SctAE	0,35	-0,51	0,045
SRrAE	0,12	-1,29	0,073
SRcdAE	0,42	2,79	0,027
SRctAE	0,21	0,33	0,066

*RMC: ressonância magnética cardíaca; VDFi: volume diastólico final indexado; SIV: septo interventricular; SrAE: strain do átrio esquerdo na sístole (função de reservatório); ScdAE: strain do átrio esquerdo na diástole precoce (função de condução); SctAE: strain do átrio esquerdo na diástole tardia (função de contração); SLG-VE: strain longitudinal global; DDFVE: diâmetro diastólico final do ventrículo esquerdo.*

### Variabilidade entre observadores e intraobservador nas medidas de deformação do AE por 2D-STE

**Figura 4:**
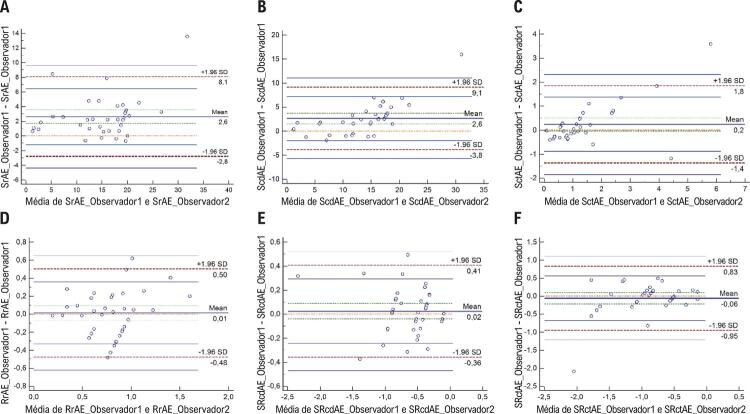
– Gráficos Bland–Altman plots para variabilidade entre observadores quanto às medidas de strain (A, B, C) e strain rate (D, E, F) do átrio esquerdo. SrAE: strain do átrio esquerdo na sístole (função de reservatório); ScdAE: strain do átrio esquerdo na diástole precoce (função de condução); SctAE: strain do átrio esquerdo na diástole tardia (função de contração); SRrAE: strain rate do átrio esquerdo na sístole (função de reservatório); SRcdAE: strain rate do átrio esquerdo na diástole precoce (função de condução); SRctAE: strain rate do átrio esquerdo na diástole tardia (função de contração).

**Figura 5:**
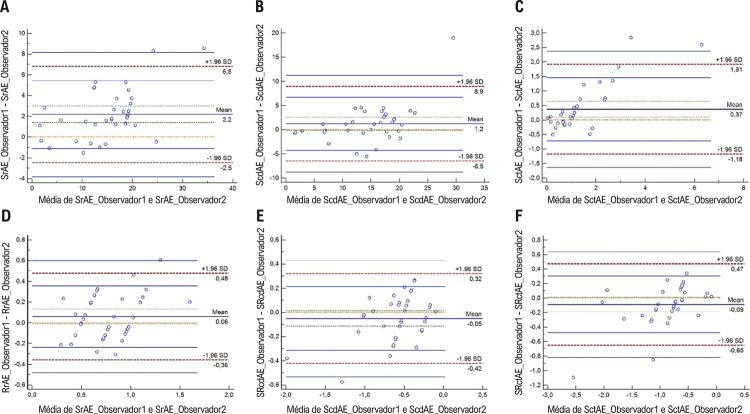
– Gráficos Bland–Altman plots para variabilidade intraobservador das medidas de strain (A, B, C) e strain rate (D, E, F) do átrio esquerdo. SrAE: strain do átrio esquerdo na sístole (função de reservatório); ScdAE: strain do átrio esquerdo na diástole precoce (função de condução); SctAE: strain do átrio esquerdo na diástole tardia (função de contração); SRrAE: strain rate do átrio esquerdo na sístole (função de reservatório); SRcdAE: strain rate do átrio esquerdo na diástole precoce (função de condução); SRctAE: strain rate do átrio esquerdo na diástole tardia (função de contração).

Os parâmetros de deformação do AE mostraram valores de coeficiente de correlação intraclasse de 0,64 a 0,94, indicando uma boa a excelente reprodutibilidade dessas medidas (Tabela suplementar). Os gráficos de Bland-Altman revelaram uma discrepância muito baixa entre observadores ( Figura 4 ) e intraobservadores ( Figura 5 ) quanto às medidas de *strain* a *strain rate* do AE.

## Discussão

O presente estudo avaliou a mecânica do AE na HVE secundária à HAS e CMH, e analisou se o remodelamento do AE esteve associado com a extensão de HVE e fibrose na CMH. Os resultados fornecem importantes informações acerca da função do AE, conforme identificado em um estudo preliminar.^19^ Conseguimos demonstrar que a mecânica do AE está diminuída tanto na HAS como na CMH. A função do AE está prejudicada na CMH ao se comparar com a HAS ( Tabela 2 , Figura 2 ). Os parâmetros que melhor discriminaram CMH foram o SRrAE e o SRctAE, com uma área sob a curva de 0,8, e um valor preditivo positivo de 86% e 88%, respectivamente. O ScdAE apresentou a maior especificidade (94%) e o SctAE a maior sensibilidade (95%) ( Tabela 3 ). Ainda, nós conseguimos demonstrar uma correlação moderada entre a mecânica do AE e o grau de fibrose avaliada por RMC nos pacientes com CHM, entre o SRcdAE e a extensão de RT pelo gadolínio ( Tabela 4 ).

O comprometimento na função de reservatório do AE é acompanhado por uma deterioração da função do VE, e é maior nos pacientes com CHM que nos pacientes com HA. A redução na função de reservatório do AE na CHM está relacionada à disfunção longitudinal do VE, devido à diminuição da sístole na base do VE, o que leva a um comprometimento no relaxamento e consequente rigidez do AE.^7 , 20^ De fato, foi demonstrada recentemente uma associação significativa entre a função de reservatório do AE (SrAE) e um pior desfecho nos pacientes com CMH, com uma correlação linear entre SrAE e níveis de peptídeo natriurético tipo-B.^21^

Apesar de a função de condução do AE estar associada à função sistólica do VE (dissincronia ventricular), ela está relacionada à extensão de hipertrofia.^2 , 20^ Em nosso estudo, o SRcdAE foi o parâmetro que melhor se correlacionou com a extensão da fibrose do VE. Tal fato deve-se provavelmente à redução da complacência do VE devido à fibrose do miocárdio, com redução da função de condução atrial na CMH.

A fase contrátil do AE também esteve comprometida na CMH, o que é de certa forma inconsistente com estudos prévios que mostraram uma tendência de aumento (embora não estatisticamente significativo) da função contrátil e de bombeamento na CMH com ausência de fibrose do VE.^2^ Isso pode estar relacionado ao fato de que 87% dos nossos pacientes com CMH já apresentavam RT com gadolínio na RMC e, assim, possível comprometimento da função contrátil do AE.

Quanto à presença de fibrose, um estudo mostrou uma correlação significativa entre o RT por gadolínio do AE e do VE na RMC, bem como um *strain* do VE anormal.^22^ Tal fato pode sugerir que a fibrose do AE seja secundária ao remodelamento do VE e aumento da pressão de enchimento. Apesar de a mecânica do AE não ter sido investigada por 2D-STE no presente estudo, podemos relacionar esses achados com nossos resultados sobre a mecânica do AE: comprometimento das funções de reservatório e contrátil, e uma correlação entre função de condução e extensão da fibrose do VE.

Apesar de a disfunção do VE, avaliada pelo SLG do VE, estar muito presente no grupo com CMH, não se observou uma relação com fibrose do VE. Outro parâmetro da disfunção do VE, o índice de performance miocárdica (IPM), também está comprometido na CMH e está relacionado com o *strain* do AE. Contudo, o IPM não foi preditivo do desfecho, diferentemente do *strain* do AE.^23^

Ao avaliar a disfunção diastólica na CMH versus HAS, observamos que o VAEi e as velocidades E e A mitral não foram diferentes entre os grupos. Tal fato demonstra a importância da mecânica do AE como um fator discriminante de miopatia do AE, a qual está comprometida mesmo antes de uma dilatação atrial esquerda. Ainda, esse dado estabelece valores basais de deformação do AE, que não sejam influenciados por outros fatores tais como FA (excluída) ou dilatação atrial esquerda importante. Com a progressão da doença, podemos esperar que a deformação do AE piora com a dilatação do AE, apesar de não termos provado isso em nosso estudo. Apesar dos diferentes valores da relação E/E’, o parâmetro não foi um bom parâmetro de discriminação entre os grupos e não se correlacionou com a extensão da fibrose miocárdica do VE na CMH. A mecânica do AE parece mais um fator discriminatório entre os grupos com HVE e está relacionada ao grau de fibrose miocárdica do VE na CMH. Esses achados também foram relatados em outras cardiomiopatias, em que não se observaram diferenças nos valores de E/E’ ou VAEi entres os três grupos de cardiomiopatia (CMH, restritiva e dilatada).^24^ Ainda, o *strain* e o *strain rate* foram melhores preditores de cardiomiopatias.^24^ Tal fato sugere que a mecânica do AE seja um marcador mais precoce tanto de disfunções atrial como miocárdica. Além disso, em pacientes com HAS e HVE significativa, nos quais a exclusão de CMH pode ser um desafio, a avaliação da mecânica do AE pode ser útil, uma vez que as três fases (de reservatório, condução e contração) não estão afetadas nesse grupo. Em nosso estudo, apesar de estatisticamente mais baixos em comparação a pacientes com CMH, os valores da espessura do SIV na nossa coorte de pacientes hipertensos ainda estavam elevados (média de 14,3±3,6 mm).

Em nosso estudo, demonstramos que o *strain* e o *strain rate* do AE são fatores discriminatórios potenciais entre CMH e HAS, não somente na resposta fisiológica à HVE como determinantes da disfunção. As implicações clínicas para o uso do strain rate do AE para o diagnóstico de CMH em pacientes com HVE ainda são incertas, uma vez que a RMC é o método de imagem mais preciso. Contudo, pudemos demonstrar que a mecânica do AE foi um fator discriminatório mais forte de HVE secundária à CMH quando comparada a outros parâmetros clássicos, como o VAEi, razão E/E’, e mesmo espessura do SIV (Tabelas 2 e 3). Ainda, observou-se uma correlação moderada entre a mecânica do AE e a extensão de fibrose do AE na CMH, o que poderia se tonar um marcador de gravidade e prognóstico em estágios iniciais ou casos duvidosos.

### Limitações

Nosso estudo tem várias limitações. Primeiro, o programa usado para a análise de *strain* foi dedicado à análise do *strain* do VE, e não do AE, o que poderia distorcer de alguma forma nossos resultados.

Segundo, a heterogeneidade da população do nosso estudo, com diferentes comorbidades, pode afetar os resultados obtidos. O fato de que uma maior proporção de pacientes hipertensos apresentou diabetes e obesidade poderia afetar a análise da mecânica do AE, devido à piora na função diastólica do VE. No entanto, essa é uma associação intrínseca que não pôde ser excluída, uma vez que há poucos pacientes que apresentassem HAS como o único fator de risco cardiovascular. A maioria dos pacientes eram adequadamente tratados com IECA e BRA, o que poderia ter afetado a remodelação do VE e compensado a disfunção sistólica nesses pacientes. Em contraste, os betabloqueadores na CMH não afetam diretamente a remodelação do VE, não influenciando, assim, a função diastólica do VE ou a mecânica do AE. No entanto, nós não pudemos avaliar se, e em que extensão tal fato tenha ocorrido. Contudo, a partir de uma comparação direta de dois grupos heterogêneos de pacientes do mundo real com HA ou CMH, podemos avaliar as detectar diferenças na mecânica do AE entre esses dois grupos, conforme observado na prática diária.

Terceiro, um estudo^25^ demonstrou que pacientes com CMH com FA paroxística podem apresentar um maior grau de miopatia do AE que pacientes sem FA. Em nosso estudo, apesar de termos excluído pacientes com FA no eletrocardiograma basal, é possível a existência de pacientes com FA paroxística que não tenham sido identificados, com um fenótipo cardíaco mais grave, que não foram totalmente caracterizados em nossa análise.

Quarto, nós não temos a validação externa dos pontos de corte propostos, de modo que não podemos sugerir propriamente o uso do *strain* do AE como um fator de discriminação preciso.

Quinto, não incluímos pacientes com HAS na análise de variabilidade, o que seria importante para avaliar realmente a reprodutibilidade de todos os parâmetros.

Finalmente, uma vez que este foi um estudo baseado em desfechos, não podemos tirar conclusões acerca do valor prognóstico do *strain rate* do AE nessa população. No entanto, este estudo tentou esclarecer a mecânica da deformação do AE na HVE secundária à CMH e à HAS. Mais estudos com um tamanho amostral maior são necessários para esclarecer o valor prognóstico do *strain rate* do AE na HVE.

## Conclusão

A mecânica do AE está globalmente comprometida na HVE secundária à HAS e à CMH. Em comparação à HAS, os melhores fatores de discriminação de CMH foram SRrAE e SRctAE. No entanto, o SRcdAE correlacionou-se melhor com o grau de fibrose do VE avaliada por RMC nos pacientes com CMH. Esses achados sugerem que a mecânica do AE pode ajudar na diferenciação de HVE entre HAS e CMH e ser um preditor potencial de gravidade da doença na CMH.

## *Material suplementar

Para informação adicional, por favor, clique aqui.


