# Supplementary material for: Mecânica Atrial na Cardiomiopatia Hipertrófica: Discriminando Hipertrofia de Fibrose Ventricular
Source: Arq Bras Cardiol. 2021 Nov 17;118(1):77–87. [Article in Portuguese] doi: 10.36660/abc.20200890 (PMC8959060; doi:10.36660/abc.20200890)
Supplement: Supplementary file 1 [file 2020-0890_supplemental_material.pdf]

Supplemental table

Inter- and intra-observer variability of left atrial strain and strain rate values

Parameter	Inter-observer variability			Intra-observer variability		
	**Bias (limits of agreement)**	**ICC (95% CI)**	**CoV (%)**	**Bias (limits of agreement)**	**ICC (95% CI)**	**CoV (%)**
LA-µsys	2.6 (-2.8-8.1)	0.91 (0.85-0.95)	17.8	2.1 (-2.5-6.8)	0.94 (0.89-0.97)	14.8
LA-µe	2.8 (-3.8-9.1)	0.87 (0.76-0.92)	21.9	1.2 (-6.5-8.9)	0.82 (0.68-0.90)	20.2
LA-µa	0.2 (-1.4-1.8)	0.79 (0.63-0.89)	22.6	0.4 (-1.2-1.9)	0.79 (0.64-0.89)	45.6
LA-SRs	0.01 (-0.5-0.5)	0.69 (0.61-0.82)	22.4	0.06 (-0.3-0.5)	0.76 (0.58-0.87)	20.2
LA-SRe	-0.02 (-0.4-0.4)	0.89 (0.81-0.94)	-21.2	-0.05 (-0.4-0.3)	0.88 (0.77-0.93)	-22.5
LA-SRa	-0.06 (-0.9-0.8)	0.64 (0.71-0-80)	-25.6	-0.08(-0.6-0.5)	0.86 (0.74-0.93)	-23.6

CI, confidence interval; CoV, coefficient of variation; ICC, intraclass correlation coefficient; LA [sys, left atrial systolic strain (reservoir function); LA [e, left atrial early diastolic strain (conduit function); LA [a, left atrial late diastolic strain (contractile function); LA SRs, left atrial systolic strain rate (reservoir function); LA SRe, left atrial early diastolic strain rate (conduit function); LA SRa, left atrial late diastolic strain rate (contractile function)
