# Supplementary material for: Mecânica Atrial na Cardiomiopatia Hipertrófica: Discriminando Hipertrofia de Fibrose Ventricular
Source: Arq Bras Cardiol. 2021 Nov 17;118(1):77–87. [Article in Portuguese] doi: 10.36660/abc.20200890 (PMC8959060; doi:10.36660/abc.20200890)
Supplement: Translation

# Translation: Atrial Mechanics in Hypertrophic Cardiomyopathy: Discriminating between Ventricular Hypertrophy and Fibrosis

**Authors:** Patrícia Marques-Alves, João André Ferreira, André Azul Freitas, José Paulo Almeida, Rui Baptista, Graça Castro, Rui Martins, Paulo Donato, Maria João Ferreira, Lino Gonçalves

**Affiliations:** 1 Portugal Departamento de Cardiologia, Centro Hospitalar e Universitário de Coimbra, Coimbra – Portugal; 2 Portugal ICBR Instituto de Investigação Clínica e Biomédica de Coimbra, Universidade de Coimbra, Coimbra – Portugal; 3 Portugal Faculdade de Medicina, Universidade de Coimbra, Coimbra – Portugal; 4 Portugal Departamento de Radiologia, Centro Hospitalar e Universitário de Coimbra, Coimbra – Portugal

**Keywords:** Cardiomyopathy, Hypertrophic, Hypertension, Echocardiography/methods, Magnetic Resonance Spectroscopy/methods, Left Ventricular Hypertrophy

## Abstract

**Background:**

Hypertrophic cardiomyopathy (HCM) and left ventricular hypertrophy (LVH) secondary to systemic hypertension (HTN) may be associated with left atrial (LA) functional abnormalities.

**Objectives:**

We aimed to characterize LA mechanics in HCM and HTN and determine any correlation with the extent of left ventricular (LV) fibrosis measured by cardiac magnetic resonance (CMR) in HCM patients.

**Methods:**

Two-dimensional speckle tracking-derived longitudinal LA function was acquired from apical views in 60 HCM patients, 60 HTN patients, and 34 age-matched controls. HCM patients also underwent CMR, with measurement of late gadolinium enhancement (LGE) extension. Association with LA strain parameters was analyzed. Statistical significance was set at p<0.05.

**Results:**

Mean LV ejection fraction was not different between the groups. The E/e’ ratio was impaired in the HCM group and preserved in the control group. LA mechanics was significantly reduced in HCM, compared to the HTN group. LA strain rate in reservoir (LASRr) and in contractile (LASRct) phases were the best discriminators of HCM, with an area under the curve (AUC) of 0.8, followed by LA strain in reservoir phase (LASr) (AUC 0.76). LASRr and LASR-ct had high specificity (89% and 91%, respectively) and LASr had sensitivity of 80%. A decrease in 2.79% of LA strain rate in conduit phase (LASRcd) predicted an increase of 1cm in LGE extension (r^2^=0.42, β 2.79, p=0.027).

**Conclusions:**

LASRr and LASRct were the best discriminators for LVH secondary to HCM. LASRcd predicted the degree of LV fibrosis assessed by CMR. These findings suggest that LA mechanics is a potential predictor of disease severity in HCM.

## Introduction

Left ventricular hypertrophy (LVH) present in hypertrophic cardiomyopathy (HCM) and arterial hypertension (HTN) is often related to myocardial dysfunction and increased risk of sudden death.^1 , 2^ In HTN, LVH occurs as a response to pressure overload, and in HCM, a complex remodeling process is initiated as a response of cardiomyocyte and noncardiomyocyte components to dynamic mechanical and neurohumoral stimuli.^1 - 3^ HCM is an autosomal-dominant disorder, associated with mutations in sarcomeric genes, affecting the atrial and ventricular myocardium.^1 , 2^

Cardiovascular magnetic resonance (CMR) allows a thorough description of HCM-related LVH and fibrosis, by means of late gadolinium enhancement (LGE).^4^ Quantitative LV LGE characterizes HCM stages, LV remodeling and systolic dysfunction and is an important predictor of sudden death.^4 , 5^ Systolic dysfunction commonly occurs in end-stage HCM, and a significant portion of patients have some extent of diastolic dysfunction.^2 , 6^

Increased LV mass and diastolic dysfunction are associated with progressive left atrial (LA) dilatation and dysfunction. Accordingly, LA remodeling is a common feature in both HCM and HTN.^2 , 7^ Furthermore, LA size and volume have been shown to be determinants of exercise capacity^8^ and of major adverse cardiac and cerebrovascular events in HCM patients.^9^

Since the LA is related to LV performance by its reservoir function during ventricular systole, conduit function during early ventricular diastole, and booster pump function during late ventricular diastole, LA myopathy could be associated with outcomes independent of LV function.^10^ LA function is correlated with heart failure symptoms in HCM and is a strong predictor for the development of atrial fibrillation (AF).^11 , 12^

The assessment of LA mechanics using two-dimensional (2D)-speckle tracking echocardiography (STE) has been shown as a feasible and reproducible marker for LA function.^13 , 14^

Although LVH seems to be the major factor of dysfunction in LV mechanics,^2^ the degree of LA dysfunction in different states of LVH (particularly, LVH secondary to HTN and to HCM) is not fully understood. LVH and fibrosis, both representing substrates of LV diastolic dysfunction, might be associated with LA dysfunction in HCM. Therefore, this study aimed (1) to characterize LA mechanics in HCM and in HTN patients with significant LVH and (2) to correlate LA function with LV fibrosis assessed by CMR in HCM patients.

## Methods

### Study population

This retrospective observational study included 60 patients diagnosed with HCM (diagnosis confirmed by CMR) and 60 HTN patients recruited from our outpatient department. We excluded patients with obstructive HCM, poor acoustic window, AF identified in the basal electrocardiogram, moderate or severe valvular disease, ischemic heart disease, or pulmonary hypertension defined as pulmonary artery systolic pressure (PASP) >45 mmHg. Patients were diagnosed with HTN about 4.2±2.3 years before. As controls, we included 34 healthy individuals without HTN, AF, or valvular disease, age-matched to HCM and HTN patients.

### Study procedures

We analyzed clinical and echocardiographic data of participants divided into the HCM group, HTN group, and control group. Data from CMR of HCM patients at the time of diagnosis were also assessed. Echocardiographic images were collected 43±18 days after HCM diagnosis was made by CMR. Echocardiographers were blinded to CMR results. The study was approved by the scientific and bioethics committees of Coimbra Hospital and University Center (Coimbra, Portugal) and was performed in accordance with the Declaration of Helsinki.

### Echocardiographic data

A complete two-dimensional echocardiographic investigation was performed in all participants, including LV- and LA-STE with global longitudinal strain (GLS) analysis. We used a Vivid 7 (GE Healthcare, Horten, Norway) cardiovascular ultrasound device, with a 1.7/3.4-MHz tissue harmonic transducer. Standard echocardiographic views were obtained with frame-rate optimization (60–80 fps in 2D imaging). We performed an offline analysis of echocardiographic data using a specific software (EchoPAC 16.0; GE Healthcare).

### LV dimensions and function

Assessment of LV size and systolic function, including measurement of LV ejection fraction (LVEF), LV end-diastolic diameter (LVDD), and LV end-systolic diameter (LVSD), followed the current recommendations.^15^ STE-derived LV-GLS was obtained using a 16-segment model of the LV.^16^ Diastolic function including mitral E velocity, mitral A velocity, and mean E/e’ ratio, was also evaluated.

### LA deformation imaging

STE-based analysis of LA mechanics was performed as previously recommended;^17 , 18^ with offline analysis of the automatically averaged longitudinal strain curves for each atrial segment by specific software^17 , 18^ ( Figure 1 ). For processing, the initial frame was chosen as the frame reflecting the P-wave onset. LA strain and strain rate during systole (LASr and LASRr, respectively), early diastole (LAScd and LASRcd), and late diastole (LASct and LASRct, respectively) were measured as indicators of the LA reservoir, conduit, and contractile functions, respectively.^14 , 18^

**Figure 1:** – Analysis of left atrial strain in controls (A) and hypertrophic cardiomyopathy (HCM) (B) patients. In controls: left atrial strain in reservoir phase (LASr) = 53%, left atrial strain in the contractile phase (LASct) = 21% and left atrial strain in the conduit phase (LAScd) (difference between LASr and LASct) = 21% (A). In HCM patients: LASr = 24%, LASct = 14% and LAScd = 10% (B)

### Inter and intra-observer variability

For analysis of inter-observer reproducibility, the 2D-STE strain and strain ratio measurements from 37 randomly selected HCM patients were made by a second investigator (JAF) and compared with those of the first observer (PMA).

The first observer repeated the measurements in the same 37 participants, and the intra-observer reproducibility was then assessed. The observers evaluated different regions of interest of the LA and were blinded to previous measurements.

### Cardiac magnetic resonance

All 60 HCM patients underwent CMR, performed with 1.5 T scanners (Philips, Best, the Netherlands) using standard protocols as suggested previously.^4^ LGE images were acquired 10-20 min after intravenous administration of gadolinium as recommended.^2^

Quantification of LGE was performed by manual adjustment of gray scale threshold, to define areas of visually identified LGE in short-axis planes and measured in centimeters (cm) of extension.

### Statistical analysis

Normality of the distribution of continuous variables was assessed by histogram analysis and the Kolmogorov–Smirnov test. Continuous variables were expressed as mean ± standard deviation, and categorical variables were expressed as frequency (percentage). Between-group differences were evaluated using one-way ANOVA. After rejecting the null hypothesis, the Bonferroni multiple comparison test was conducted. For each variable with non-normal distribution, the homogeneity of variance was assessed using the Levene test. For categorical variables, the chi-square test or the Fisher’s exact test was used, as appropriate. Linear regression was used to correlate several continuous parameters. Assumptions for linear regression were checked beforehand. Namely, a linear relationship between data was visually assessed using scatter plots; no outliers were detected; autocorrelation was excluded using the Durbin-Watson test; homogeneity of variance of the residuals (homoscedasticity) was also checked visually by plotting residuals versus fitted values. Analysis of receiver operating characteristic (ROC) curves was performed to compute the discriminative power of LA mechanics parameters in HCM versus HTN. The curves were compared using the Delong method. We used the Bland–Altman method, intraclass correlation coefficient, and coefficient of variation to assess the inter- and intra-observer variability of LA 2D-STE strain and SR measurements. Statistical significance was set at p-<0.05. Stata IC for Windows (version 13; StataCorp, Lakeway Drive, TX, USA) and MedCalc for Windows (version 14.8.1; MedCalc Software, Ostend, Belgium) were used for the statistical analyses.

Regarding sample size, we planned a study of a continuous response variable in independent control and experimental subjects, with 0.5 control(s) per experimental subject. In LA mechanics analysis (LA strain in reservoir and contractile phases), the desirable response within each subject group is normally distributed with a standard deviation of five.^7 , 10^ If the true difference between experimental and control subject means is four, we would need 51 experimental subjects and 26 control subjects to be able to reject the null hypothesis that the population means of the experimental and control groups are equal with a probability (power) of 0.9. The type I error probability associated with this test of this null hypothesis is 0.05. We could further include 60 HCM patients and 60 HTN patients to further improve the statistical power of the study.

## Results

### Study population

The clinical features of the study population are summarized in Table 1 . The mean age of HCM patients was 55±18 years, and 57% of patients were male. This did not vary significantly from HTN patients and controls. HTN patients had more diabetes *mellitus* , dyslipidemia, and obesity.

**Table 1:** – Demographic and clinical characteristics of patients the study population

Characteristic	Controls (n=34)	HCM group (n=60)	HTN group (n=60)	p-value
Age, years (±SD)	56±10	55±18	61±12	0.081*
Male sex (%)	20 (55)	34 (57)	34 (57)	0.124^#^
Diabetes *mellitus* (%)	0	15 (25)	35 (58)	0.022^#^
Dyslipidemia (%)	0	24 (40)	43 (72)	0.014^#^
Obesity (%)	0	12 (20)	28 (47)	0.031^#^
**Antihypertensive drugs**				
Diuretics use (%)	0	15 (25)	32 (53)	0.018^#^
Beta-blocker use	0	45 (75)	37 (62)	0.068^#^
ACEI/ARB use	0	41 (68)	45 (75)	0.072^#^
CCB use	0	27 (45)	42 (70)	0.032^#^
Other anti-HTN use	0	12 (20)	20 (34)	0.056^#^

Data are given as mean ± standard deviation or as frequency (percentage). ACEI/ARB: angiotensin converting enzyme inhibitor/angiotensin receptor blockers; CCB: calcium channel blocker; HCM: hypertrophic cardiomyopathy; HTN: arterial hypertension. * One-Way ANOVA.^#^Chi-square test.

In the HCM group, there was a higher use of beta-blocker therapy, while in the HTN group, a higher prescription of angiotensin-converting enzyme inhibitors (ACEI) and angiotensin receptor blockers (ARB) was found.

### Conventional echocardiographic parameters

The echocardiographic characteristics of participants are summarized in Table 2 . The mean LVEF were not different between the groups. LVDD was higher in the HTN group than in the HCM group. PASP was not different between the HCM and HTN groups and was significantly reduced in the control group.

**Table 2:** – Echocardiographic parameters of the study population

Parameters	Controls	HTN group	HCM group	Global P-value	p value: controls vs HTN	p value: HCM vs HTN
LVEF, %	62.9±4.3	62.9±4.9	66.5±10.1	0.083	0.969	0.055
LVDD, mm	48.3±5.2	51.9±0.8	49.4±1.0	0.108	0.019	0.083
LVSD, mm	30.3±3.2	32.3±0.7	30.7±0.9	0.369	0.119	0.225
IVS, mm	10.2±2.8	14.3±3.6	16.5±5.4	0.028	<0.001	0.032
LV-GLS, %	-20.6±1.1	-17.5±0.7	-12.7±0.5	<0.001	0.192	0.008
PASP, mmHg	22.1±4.7	26.3±0.2	28.6±1.3	0.021	0.009	0.245
LAVi, mL/m^2^	23.5±4.2	31.1±1.3	33.5±2.5	<0.001	0.001	0.067
Mitral E velocity, m/s	0.8±0.1	0.7±0.2	0.8±0.2	0.156	0.068	0.182
Mitral A velocity, m/s	0.5±0.1	0.8±0.2	0.7±0.3	0.005	<0.001	0.151
E/e’ ratio	7.0±1.65	13.2±1.2	16±1.0	<0.001	<0.001	0.035
LASr, %	36.9±10.8	24.4±8.2	17.2±9.0	<0.001	<0.001	<0.001
LAScd, %	25.9±13.3	19.9±8.7	15.4±9.1	<0.001	0.067	0.022
LASct, %	10.9±6.2	5.1±0.9	1.9±0.3	<0.001	0.003	<0.001
LASRr, %	1.9±0.5	1.2±0.1	0.8±0.1	<0.001	<0.001	<0.001
LASRcd, %	-2.1±0.6	-1.8±0.1	-0.6±0.1	<0.001	0.082	<0.001
LASRct, %	-1.9±0.7	-1.7±0.1	-0.9±0.1	<0.001	0.344	<0.001

*Statistical analysis: one-way ANOVA with Bonferroni test for multiple comparisons. HCM: hypertrophic cardiomyopathy; HTN: hypertension; IVS: interventricular septum; LASr: left atrial systolic strain (reservoir function); LAScd: left atrial early diastolic strain (conduit function); LASct: left atrial late diastolic strain (contractile function); LASRr: left atrial systolic strain rate (reservoir function); LASRcd: left atrial early diastolic strain rate (conduit function); LASRct: left atrial late diastolic strain (contractile function); LAVi: left atrial volume indexed; LVDD: left ventricular end-diastolic diameter; LVSD: left ventricular end-systolic diameter; LVEF: left ventricular ejection fraction; LV-GLS: left ventricular global longitudinal strain; PASP: pulmonary artery systolic pressure.*

Regarding LV diastolic function, mitral E velocity did not vary among groups, and mitral A velocity was higher lower in the HTN group than in the control group. E/e’ ratio was significantly impaired in the HCM group and preserved in the control group.

### LA function

Compared to controls, HCM and HTN patients had significantly larger LA volume indexed to the body surface area (LAVi) ( Table 2 ). LA deformation parameters were globally decreased in both HTN and HCM groups, in relation to the control group. In the HTN group, reservoir function was preserved, although significantly reduced compared to that in the control group; the conduit phase was not different from the control group, and strain in contractile phase (but not LASRct) was significantly impaired in HTN patients ( Table 2 , Figure 2 ).

**Figure 2:** – Left atrial deformation parameters in patients with hypertrophic cardiomyopathy (HCM), arterial hypertension (HTN), and controls; strain (A) and strain rate (D) in the reservoir phase; strain (B) and strain rate (E) in the conduit phase; strain (C), and strain rate (E) in the contractile phase. LASr: left atrial systolic strain (reservoir function); LAScd: left atrial early diastolic strain (conduit function); LASct: left atrial late diastolic strain (contractile function); LASRr: left atrial systolic strain rate (reservoir function); LASRcd: left atrial early diastolic strain rate (conduit function); LASRct: left atrial late diastolic strain (contractile function).

All LA deformation phases were significantly reduced in the HCM group than in the HTN group ( Table 2 , Figure 2 ). Of all LA mechanics parameters, LASRr and LASRct were the best discriminators of HCM (versus HTN), followed by LASr. LASRr and LASRct had high specificity and positive predictive value (PPV) ( Table 3 ). Despite the discriminative power, LAScd had the highest specificity (94%) and LASct the highest sensitivity (95%) ( Table 3 ).

**Table 3:** – Discriminative power of echocardiographic parameters (HCM vs HTN groups)

	AUC	95% CI	p-value	Sensitivity (%)	Specificity (%)	Criterion	PPV (%)	NPV (%)
LASr(%)	0.76	0.66-0.84	<0.001	80	71	21.8	73	78
LAScd (%)	0.65	0.54-0.74	0.012	32	94	9.9	84	58
LASct(%)	0.65	0.54-0.75	0.016	95	34	5.1	59	87
LASRr (%)	0.80	0.71-0.88	<0.001	65	89	0.8	86	72
LASRcd (%)	0.69	0.59-0.79	<0.001	54	87	-0.8	81	65
LASRct (%)	0.80	0.71-0.88	<0.001	64	91	-0.9	88	72
IVS (mm)	0.62	0.51-0.70	0.012	55	74	15.2	68	63
LV-GLS (%)	0.74	0.64-0.83	<0.001	57	84	-13.5	78	66
E/e’ ratio	0.67	0.55-0.78	0.009	67	71	13	70	68

*AUC: area under the curve; HTN: arterial hypertension; HCM: hypertrophic cardiomyopathy; IVS: interventricular septum; LASr: left atrial systolic strain (reservoir function); LAScd: left atrial early diastolic strain (conduit function); LASct: left atrial late diastolic strain (contractile function); LASRr: left atrial systolic strain rate (reservoir function); LASRcd: left atrial early diastolic strain rate (conduit function); LASRct: left atrial late diastolic strain (contractile function); LV-GLS: left ventricular global longitudinal strain; NPV: negative predictive value; PPV: positive predictive value.*

### CMR parameters

All HCM patients underwent CMR (the gold standard method for diagnosis). The mean indexed end-diastolic volume (EDVi) was 96±32 mL/m^2^ , the mean interventricular septum thickness (IVS) was 18.7±3.5 mm. Approximately 34% of the patients had systolic anterior movement of the mitral valve, and 12% of the patients had apical HCM. LGE was present in 52 (87%) HCM patients, and the mean area of extension was 2.8 cm.

### CMR and echocardiographic parameters

Table 4 summarizes the results of the linear regression analysis between the extension of LGE (in cm) and several CMR and echocardiographic parameters in HCM patients. LV measures, EDVi (by CMR), LVDD and LVSD by echocardiography, LV-GLS or the E/E’ ratio did not predict LGE extension. IVS thickness measured by CMR, but not by echocardiography, predicted LGE. Both LASr and LASRcd predicted LGE extension. A decrease of 0.5% in LASr and of 2.79% in LASRcd predicted an increase of 1cm in LGE ( Figure 3 ).

**Table 4:** – Linear regression analysis between the extension of late gadolinium enhancement (in cm) and several CMR and echocardiographic parameters

Late gadolinium enhancement	Adj R^2^	β	p-value
IVS by CMR	0.32	0.12	0.051
IVS by echocardiography	0.24	0.08	0.088
EDVi by CMR	0.01	0.01	0.843
LVDD	0.01	-0.02	0.795
E/E’ ratio	0.01	-0.04	0.802
LV-GLS	0.04	0.08	0.467
LASr	0.12	-0.02	0.085
LAScd	0.15	-0.01	0.092
LASct	0.35	-0.51	0.045
LASRr	0.12	-1.29	0.073
LASRcd	0.42	2.79	0.027
LASRct	0.21	0.33	0.066

*CMR: cardiac magnetic resonance; EDVi: indexed end-diastolic volume; IVS: interventricular septum; LASr: left atrial systolic strain (reservoir function); LAScd: left atrial early diastolic strain (conduit function); LASct: left atrial late diastolic strain (contractile function); LASRr: left atrial systolic strain rate (reservoir function); LASRcd: left atrial early diastolic strain rate (conduit function); LASRct: left atrial late diastolic strain (contractile function); LGE: late gadolinium enhancement; LVDD: left ventricular end-diastolic diameter; LV-GLS: left ventricular global longitudinal strain.*

**Figure 3:** – Linear correlation between late gadolinium enhancement (LGE) extension and interventricular septum thickness measured by cardiac magnetic resonance (A), left atrial late diastolic strain (contractile function) (LASct) (B) and left atrial early diastolic strain rate (conduit function) (LASRcd) (C).

### Inter- and intra-observer variability in 2D-STE measurements of LA deformation

The parameters of LA deformation showed intraclass correlation coefficient values of 0.64 to 0.94, indicating good to excellent reproducibility of such measurements (Supplemental Table). The Bland–Altman plots revealed very small inter-observer ( Figure 4 ) and intra-observer ( Figure 5 ) discrepancy in the measurements of LA strain and strain rate.

**Figure 4:** – Bland–Altman plots for inter-observer variability of left atrial strain (A, B, C) and strain rate (D, E, F) measurements. LASr: left atrial systolic strain (reservoir function); LAScd: left atrial early diastolic strain (conduit function); LASct: left atrial late diastolic strain (contractile function); LASRr: left atrial systolic strain rate (reservoir function); LASRcd: left atrial early diastolic strain rate (conduit function); LASRct: left atrial late diastolic strain (contractile function).

**Figure 5:** – Bland–Altman plots for intra-observer variability of left atrial strain (A, B, C) and strain rate (D, E, F) measurements. LASr: left atrial systolic strain (reservoir function); LAScd: left atrial early diastolic strain (conduit function); LASct: left atrial late diastolic strain (contractile function); LASRr: left atrial systolic strain rate (reservoir function); LASRcd: left atrial early diastolic strain rate (conduit function); LASRct: left atrial late diastolic strain (contractile function).

## Discussion

This study examined LA mechanics in LVH secondary to HTN and HCM and analyzed if LA remodeling was associated with the extent of LV hypertrophy and fibrosis in HCM. The results provide insights into LA function, as identified in a preliminary study.^19^ We could demonstrate that LA mechanics is globally decreased in both HTN and HCM. LA function is significantly impaired in HCM in relation to HTN ( Table 2 , Figure 2 ). The best discriminating factors of HCM were LASRr and LASRct with an area under the curve of 0.8 and a positive predictive value of 86% and 88%, respectively. LAScd had the highest specificity (94%) and LASct the highest sensitivity (95%) ( Table 3 ). Furthermore, we could demonstrate a moderate correlation between LA mechanics and the degree of LV fibrosis assessed by CMR in HCM, namely, LASRcd and extension of LGE ( Table 4 ).

LA reservoir function impairment is accompanied by deterioration of LV function, and is greater in HCM than in HTN. The reduction in LA reservoir function in HCM is related to the LV longitudinal dysfunction, due to a reduction in the systolic descent of LV base, which leads to an impaired LA relaxation and stiffness.^7 , 20^ Indeed, it has recently been reported a significant association between LA reservoir function (LASr) and worse outcome in HCM patients, with a linear correlation between LASr and B-type natriuretic peptide levels.^21^

Although LA conduit function is associated with LV systolic function (ventricular desynchrony), it is less related to the extent of hypertrophy.^2 , 20^ In our study, LASRcd was the parameter with the best correlation with the extent of LV fibrosis. This is probably explained by reduction of LV compliance due to myocardial fibrosis, with accompanying reduction of the atrial conduit function in HCM.

LA contractile phase was also impaired in HCM, which is somewhat inconsistent with previous reports that showed a trend toward increased LA contractile booster pump function in HCM with absent LV fibrosis (although not statistically significant).^2^ This might be related to the fact that 87% of our HCM patients already presented with LGE on CMR, so LA contractile function might already be compromised.

Regarding the presence of fibrosis, one study showed a significant correlation between LA and LV LGE on CMR, as well an abnormal LV strain.^22^ This might suggest that LA fibrosis is secondary to LV remodeling and increased filling pressure. Although LA mechanics by 2D-STE was not performed in this study, we could relate these findings to our results on LA mechanics: impaired reservoir and contractile functions, and correlation between conduit function and the extent of LV fibrosis.

Although LV disfunction, assessed by LV-GLS, was very present in the HCM cohort, it was not related to LV fibrosis. Another parameter of LV disfunction, the myocardial performance index (MPI), is also impaired in HCM and is related to LA strain. However, MPI was not predictive of outcome, in contrast to LA strain.^23^

When evaluating diastolic dysfunction in HCM versus HTN, we noted that LAVi and mitral E and A velocities were not different between the groups. This fact demonstrates the importance of LA mechanics as a discriminator of LA myopathy, that is impaired even before significant LA dilation. Also, it establishes LA deformation baseline values, that are not influenced by other factors such as AF (excluded) or significant LA dilatation. With progression of disease, we can expect that LA deformation worsens with LA dilation, however we could not prove this in our study. Despite the differences in the E/E’ ratios values, the parameter was not a good discriminator between groups and was not correlated to the extent of LV myocardial fibrosis in HCM. LA mechanics appears more as discriminator between LVH groups and is related to the degree of LV myocardial fibrosis in HCM. These findings have also been described in other cardiomyopathies, in which no differences were found in E/E’ and LAVi values among three cardiomyopathy groups (HCM, restrictive and dilated).^24^ LA strain and strain rate values had a significant progressive decrease along the groups (HCM, restrictive and dilated). Also, LA strain and strain rate were the best discriminators of cardiomyopathies.^24^ This suggests that LA mechanics might be an earlier marker of both atrial and myocardial dysfunctions. Furthermore, in HTN patients with significant LVH, in whom excluding HCM might be challenging, the evaluation of LA mechanics might be useful, since the three (reservoir, conduction and contractile) phases are not very impaired in this group. In our study, despite statistically lower as compared with HCM patients, values of IVS thickness in our HTN cohort were still high (mean of 14.3±3.6 mm).

In the present study, we demonstrated that LA strain and strain rate were potential discriminators between HCM and HTN, not only in physiologic response to LVH, but also to the determinants of dysfunction. The clinical implications for the use of LA strain rate imaging for diagnosis of HCM in patients with LVH remains uncertain, since CMR is the most accurate imaging method. Nevertheless, we could demonstrate that LA mechanics was a stronger discriminator of LVH secondary to HCM, when compared to other classic parameters, such as LAVi, E/E’ ratio, and even IVS thickness (Tables 2 and 3). In addition, LA mechanics was moderately correlated to LV fibrosis extension in HCM, which could potentially become a marker of severity and prognosis in earlier stages or doubtful cases.

### Limitations

Our study has several limitations. First, the software used for strain analysis was dedicated to the analysis of the LV strain, and not to LA strain, which could distort our results to some extension.

Second, the heterogeneity of our study population in terms of different comorbidities can affect the obtained results. The fact that a higher proportion of HTN patients had diabetes and obesity could affect the analysis of LA mechanics, due to a worsening in the LV diastolic function. However, this is an intrinsic association that could not be excluded, as there are few patients with HTN as the only cardiovascular risk factor. Most patients were adequately treated with ACEI and ARB, which could counteract the diastolic dysfunction in these patients, by affecting LV remodeling. In contrast, beta-blockers in HCM does not directly affect LV remodeling, without necessary changes in LV diastolic function or LA mechanics. However, we could not evaluate if and to what extent this was true. Nevertheless, by directly comparing two heterogenous groups of real-world patients with HTN or HCM, we could detect differences in LA mechanics between these two groups as seen in everyday practice.

Third, it has been demonstrated that HCM patients with paroxysmal AF might have a greater degree of LA myopathy than patients without AF.^25^ In our study, although we excluded patients with AF on basal electrocardiogram, there may still exist unidentified patients with paroxysmal FA, with a more severe cardiac phenotype, that were not entirely characterized in our analysis.

Fourth, we did not have external validation for the proposed cutoffs, so we could not properly suggest LA strain as an accurate discriminator.

Fifth, we did not include HTN patients in the variability analysis, which would be important to truly evaluate reproductivity of all parameters.

Finally, this was not an outcome-based study, so we could not draw any conclusions about the prognostic value of LA strain rate in this population. Nevertheless, this study attempted to clarify the LA deformation mechanics in LVH secondary to HCM and to HTN. Future studies with larger samples are warranted to clarify the prognostic value of LA strain rate in LVH.

## Conclusion

LA mechanics is globally impaired in LVH secondary to HTN and to HCM. Compared to HTN, the best discriminators of HCM were LASRr and LASRct. However, LASRcd was better correlated with the degree of LV fibrosis assessed by CMR in HCM patients. These findings suggest that LA mechanics can help differentiating LVH between HTN and HCM and is a potential predictor of disease severity in HCM.

## *Supplemental Materials

For additional information, please click here.
